# The trends of antihypertensive drug prescription based on the Japanese national data throughout the COVID-19 pandemic period

**DOI:** 10.1038/s41440-024-01706-7

**Published:** 2024-06-03

**Authors:** Shotaro Natsume, Michihiro Satoh, Takahisa Murakami, Masato Sasaki, Hirohito Metoki

**Affiliations:** 1 Division of Public Health, Hygiene and Epidemiology, Tohoku Medical and Pharmaceutical University, Sendai, Japan; 2 Division of Infection and Host Defense, Faculty of Pharmaceutical Sciences, Tohoku Medical and Pharmaceutical University, Sendai, Japan; 3 Department of Pharmacy, Tohoku Medical and Pharmaceutical University Hospital, Sendai, Japan; 4 Department of Preventive Medicine and Epidemiology, Tohoku Medical Megabank Organization, Tohoku University, Sendai, Japan

**Keywords:** Hypertension, Blood pressure, Antihypertensive agents, Angiotensin-converting enzyme inhibitors, Angiotensin receptor antagonists, Epidemiology

## Abstract

In 2020, concerns arose about the potential adverse effects of angiotensin II type 1 receptor blockers (ARBs) and angiotensin-converting enzyme inhibitors (ACEIs) on patients with the Coronavirus Disease 2019 (COVID-19). However, there is no national data on antihypertensive prescriptions during the COVID-19 pandemic in Japan. This study aimed to explore the trends in antihypertensive drug prescriptions in Japan throughout COVID-19 pandemic period. This study used data from the National Database (NDB) Open Data in Japan, an annual publication by the Ministry of Health, Labour and Welfare. To capture changes before and after social activity restrictions, the present study focused on extracting the number of prescribed oral medicine tablets for outpatients from the NDB Open Data from 2018 to 2021. The fiscal year 2020 exhibited the lowest for both outpatient claims and prescribed drugs. In contrast, all categories of antihypertensive drug prescription showed annual increases, and no specific changes in the prescription patterns of ARBs and ACEIs around fiscal year 2020 were observed. This study implies that antihypertensive drug prescriptions were adequately maintained throughout the COVID-19 pandemic in Japan.

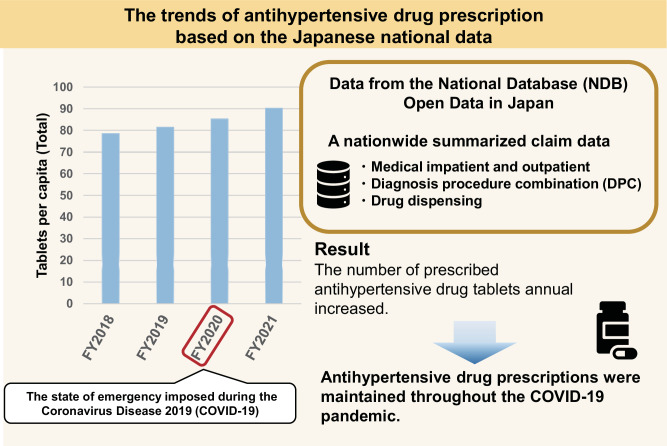

## Introduction

Systolic and diastolic blood pressure (BP) levels were reported to  increase by 1–2 mmHg and 0.4–1 mmHg, respectively, in response to the state of emergency imposed during the Coronavirus Disease 2019 (COVID-19) pandemic in Japan. This elevation was more pronounced among patients undergoing treatment for hypertension than untreated individuals. Notably, the increase remained significant even after adjusting for factors such as age, body mass index (BMI), alcohol consumption, smoking, medical history, and physical activity [1]. A similar trend was observed in a study conducted in the United States, where systolic and diastolic BP increased by 1.5–2 mmHg and 1.1–1.5 mmHg, respectively, following social activity restrictions [2]. However, both reports did not indicate a direct factor responsible for the rise in BP after social activity restrictions. An earlier study in Japan [1] reported that changes in antihypertensive drug prescriptions might have substantially contributed to the observed BP increase post-social activity restriction.

In 2020, concerns arose about the potential adverse effects of angiotensin II type 1 receptor blockers (ARBs) and angiotensin-converting enzyme inhibitors (ACEIs) on patients with COVID-19 [3]. Scientific organisations issued position statements or communications supporting the continued use of ARBs or ACEIs in stable patients with COVID-19 [4]. However, a previous study based on a Japanese hospital outpatient database revealed a tendency for a decline in the proportion of prescriptions for ARBs, a type of antihypertensive medication, immediately following the state of emergency [5]. Although some reports indicated no change in the number of ARBs prescriptions for patients with heart failure [6], there is no national data on antihypertensive prescriptions during the COVID-19 pandemic. Therefore, it becomes crucial to clarify how prescriptions for antihypertensive drugs, including ARBs and ACEIs, changed during the COVID-19 pandemic using a database that includes a diverse population of patients undergoing antihypertensive treatment in Japan. To address this gap, this study aimed to explore the trends in antihypertensive drug prescriptions in Japan throughout the COVID-19 pandemic period.

Point of view

- **Clinical relevance:**
  The prescriptions of major antihypertensive drug classes showed annual increases, and no specific changes in the prescription patterns of angiotensin II type 1 receptor blockers (ARBs) and angiotensin-converting enzyme inhibitors (ACEIs) around fiscal year 2020 were observed.
- **Future direction:**
  This study used summary statistics from the National Database Open Data in Japan. Individual data are needed for detailed tests of change and analyses of the factors driving prescription changes.
- **Consideration for the Asian population:**
  ARBs and ACEIs prescriptions could have been adequately maintained throughout the coronavirus disease 2019 pandemic in Japan despite initial speculations regarding the potential adverse impacts of ARBs and ACEIs on COVID-19 patients.

## Methods

This study used data from the National Database (NDB) Open Data in Japan, an annual publication by the Ministry of Health, Labour and Welfare [7]. The most recent release, the 8th NDB Open Data, became available in August 2023. The NDB compiles two main types of data, including specific health checkup data. The drug claims data used in this study were derived from information encompassing medical inpatient and outpatient claims, diagnosis procedure combination (DPC) claims, and drug dispensing.

Within the NDB Open Data, prescription quantities were represented by the total number of tablets prescribed (mostly also dispensed) per drug product during a fiscal year. To capture changes before and after social activity restrictions, the study focused on extracting the number of prescribed oral medicine tablets for outpatients from the NDB Open Data from 2018 to 2021.

To estimate the number of prescribed tablets per capita, we calculated by dividing the number of prescribed tablets within the study period by the corresponding population data for each year. According to the Japanese Society of Hypertension guidelines [8], we defined calcium channel blockers (CCBs), ARBs, ACEIs, β (α/β)-blockers, thiazide diuretics (TZD), and others (including mineralocorticoid receptor antagonists, loop diuretics, and alpha-adrenergic blocking agents), as the main antihypertensive agents. Combination drugs were treated as independent counts for each component. The prescribed number of tablets per individual was then tabulated on a nationwide scale and further stratified by region such as Hokkaido/Tohoku, Kanto, Chubu, Kansai, Chugoku/Shikoku, and Kyushu/Okinawa.

## Results

The fiscal year 2020, spanning from April 2020 to March 2021, exhibited the lowest figures for both outpatient claims and prescribed drugs (Table 1). In contrast, the number of prescribed tablets for antihypertensive drugs demonstrated an upward trend from fiscal year 2018 to fiscal year 2021. In 2018, the highest number of prescribed tablets was recorded for CCBs, followed by ARBs, β (α/β)-blockers, TZD, and ACEIs. Notably, all categories of prescribed medicine showed annual increases, and no specific changes in the prescription patterns of ARBs and ACEIs around fiscal year 2020 were observed (Table 1).

**Table 1:** Changes in antihypertensive drug prescriptions and dispenses

Variables	FY2018	FY2019	FY2020	FY2021
Total population of Japan, *n* [per 1000] Population	124,218	123,731	123,399	122,780
No. of medical outpatient claims *n* [per1000]	1,006,500	1,005,200	916,500	967,300
No. of drug dispensing *n* [per 1000]	657,500	662,300	611,500	644,200
No. of tablets prescribed [in 1000,000 units] (Difference from the previous FY)				
CCBs	4,524	4712 (188)	4920 (208)	5207 (287)
ARBs	2,640	2697 (57)	2796 (99)	2963 (167)
ACEIs	114	118 (4)	122 (4)	134 (12)
β (αβ)-blockers	1,260	1334 (74)	1404 (70)	1463 (59)
TZD	325	312 (−13)	343 (31)	355 (12)
Others	892	908 (16)	940 (32)	961 (21)
Total	9,756	10,083 (327)	10,526 (443)	11,084 (558)
No. of tablets (per capita)				
CCBs	36.4	38.1 (1.7)	39.9 (1.8)	42.4 (2.5)
ARBs	21.3	21.8 (0.5)	22.7 (0.9)	24.1 (1.4)
ACEIs	0.9	1.0 (0.1)	1.0 (0.0)	1.1 (0.1)
β (αβ)-blockers	10.1	10.8 (0.7)	11.4 (0.6)	11.9 (0.5)
TZD	2.6	2.5 (−0.1)	2.8 (0.3)	2.9 (0.1)
Others	7.2	7.3 (0.1)	7.6 (0.3)	7.8 (0.2)
Total	78.5	81.5 (3.0)	85.3 (3.8)	90.3 (5.0)

The National Database (NDB) Open Data includes 100 products listed in descending order of prescribed tablets within each efficacy category. Therefore, this study calculated the top 100 drugs with the highest prescription numbers in each efficacy category. Other efficacy categories include mineralocorticoid receptor antagonists, loop diuretics, and alpha-adrenergic blocking agents

*CCBs* calcium channel blockers, *ACEIs* angiotensin-converting enzyme inhibitors, *ARBs* angiotensin II type 1 receptor blockers, *TZD* thiazide (-like) diuretics, *β (αβ)-blockers* Beta (alpha and beta) adrenergic blocking agent, *FY* fiscal year

**Fig. 1:**
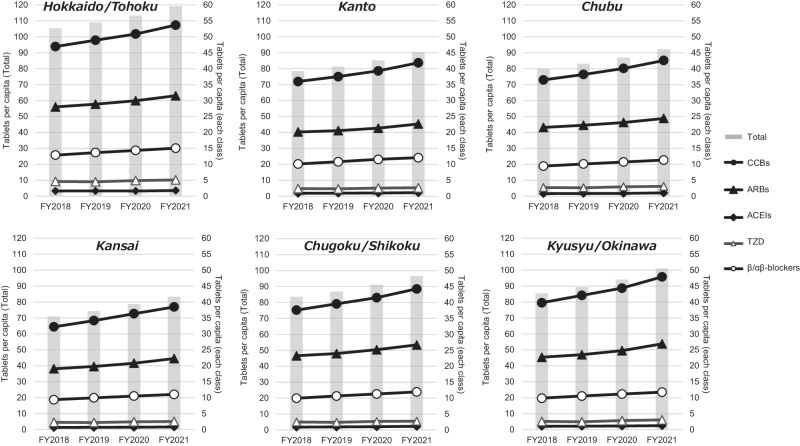
Trends in antihypertensive drug prescriptions per capita across Japanese regions. The left vertical axis represents total antihypertensive drugs, while the right vertical axis is dedicated to each antihypertensive drug class. Both units are expressed as “prescribed tablets per capita”

We further stratified the data based on Japanese regions. The Hokkaido/Tohoku regions displayed a notably higher number of prescribed antihypertensive drug tablets, while no specific regional variations were observed in the annual trends of antihypertensive drug prescriptions (Fig. 1).

## Discussion

The number of both outpatient claims and prescribed drugs decreased from fiscal year 2018 to fiscal year 2021, especially in fiscal year 2020. A study reported a substantial decrease in the number of visiting outpatients and outpatient prescriptions, particularly in May 2020 compared to May 2019 [9]. In our study, while the number of claims temporarily decreased from 2019 to 2020, the number of prescribed antihypertensive drug tablets showed a consistent upward trend. This trend remained consistent for both national averages and regional categories. Additionally, the prescribing trends for ARBs and ACEIs did not change significantly during the COVID-19 pandemic. This may be supported by the observation that the prescribing trends for antihypertensive drugs were similar across regions. Consequently, it appears that the increase in BP observed in 2020 was not attributable to antihypertensive drugs, but rather might be influenced by factors such as medication adherence and mental stress. The length of prescription coverage used in patients with chronic conditions increased throughout the COVID-19 pandemic period in Japan [9]. This may have negatively impacted medication adherence although few studies assessed medication adherence during the pandemic in Japan. Regarding mental stress, depression and anxiety disorders has been reported to increase during the COVID-19 pandemic in most countries [10].

A study reporting a decreasing trend in ARBs prescription rates after the COVID-19 pandemic [5] used the Medical Data Vision (MDV) database, which includes the DPC hospitals as survey data. This survey highlighted declining ARBs prescription rates even before 2019, a trend inconsistent with our current findings. Since the DPC system in Japan primarily serves acute-care hospitals, the previous report was limited to antihypertensive prescription trends among patients with relatively severe diseases [5, 6]. Another study reported that most of Japanese hypertensive patients prefer visiting primary clinics rather than acute-care hospitals [11]. Our study, based on the NDB Open Data, encompassing nationwide claims, showed no decrease in the number of prescribed ARBs and ACEIs tablets post-COVID-19 pandemic. This implies potential differences in antihypertensive drug prescription trends between data from DPC hospitals and those from primary clinics. Several scientific societies have issued statements calling for ARBs and ACEIs should be continued in COVID-19 patients because the benefits outweigh the risks [4]. In our current study, the results did not show decrease in the number of prescribed ARBs and ACEIs tablets post-COVID-19 pandemic. This result shows that ARBs and ACEIs prescriptions could have been adequately maintained throughout the COVID-19 pandemic in Japan. Although one report indicated the positive association between loop prescription and COVID-19 risk, it is thought that the use of loop diuretics only reflects the existence of impaired conditions such as heart failure or renal damage [12].

Significant regional differences were observed in the number of prescribed antihypertensive drugs per capita. The findings indicate that antihypertensive prescriptions in the Hokkaido/Tohoku region are substantially higher than the national average. This discrepancy may be attributed to regional variations in the aging population, the prevalence of hypertensive patients, and differences in criteria for reviewing medical claims by insurers. Notably, this result could not be precisely adjusted for age, which limits our ability to conduct a detailed causal analysis.

In our study, the number of prescribed tablets for antihypertensive drugs showed annual increases throughout the COVID-19 pandemic period. This is thought to be mainly due to the aging of the population. As for other factors, improved awareness of the importance of hypertension treatment may have influenced the increase in antihypertensive prescriptions [13, 14].

This study has some potential limitations. First, the NDB Open Data only provided information on the number of tablets prescribed in each fiscal year and did not include data on the number of patients with hypertension who received prescriptions of antihypertensive drugs. Second, the NDB Open Data offered data solely on the 100 most prescribed drug products within each therapeutic category. Consequently, this study could not include the number of prescriptions that were not prescribed in the top 100, potentially leading to an underestimation of the total number of antihypertensive drug tablets prescribed in each fiscal year. Individual data are needed for detailed tests of change and analyses of the drivers of prescribing change.

## Perspective of Asia

The number of antihypertensive prescriptions may have increased in Asia between 2018 and 2021. In addition, without being influenced by initial speculations about the potential adverse effects of ARBs and ACEIs on COVID-19 patients, physicians may have appropriately prescribed antihypertensive medications to prevent patients from running out of medication during the COVID-19 pandemic, at least in Japan.

## Conclusion

Based on the NDB Open Data, there was no evident decline in the number of prescribed antihypertensive drug tablets, including ARBs and ACEIs, was observed during or after the COVID-19 pandemic. This implies that antihypertensive prescriptions were adequately maintained throughout the COVID-19 pandemic in Japan.
